# Examining the relationship between breastfeeding duration at age two and beyond with parenting styles and infant feeding attitudes in Turkey (2023–2024)

**DOI:** 10.1590/1806-9282.20251739

**Published:** 2026-06-15

**Authors:** Şenay Türe, İlknur Münevver Gönenç, Sevtap Velipaşaoğlu

**Affiliations:** 1 Akdeniz University, School of Medicine, Department of Pediatrics, Division of Social Pediatrics – Antalya, Turkey.; 2 Ankara University, Faculty of Nursing – Ankara, Turkey.

**Keywords:** Breastfeeding, Exclusive, Parenting, Infant nutrition, Time factors

## Abstract

**OBJECTIVE:**

Exclusive breastfeeding for 6 months and continued breastfeeding for 24 months remain low due to various challenges. The aim of this study was to examine the impact of mothers’ infant feeding attitudes and parenting styles, as modifiable factors, on breastfeeding duration.

**METHODS:**

This cross-sectional descriptive study was conducted at Akdeniz University Faculty of Medicine Hospital between April 1, 2023, and April 1, 2024. The sample comprised 224 mothers of children aged 2–3 years who attended the child health follow-up clinic. Data were obtained through face-to-face interviews using the Introductory Information Form, Child Nutrition Questionnaire, Iowa Infant Feeding Attitudes Scale, and Parenting Styles Scale. Group differences were examined with t-tests and analysis of variance, correlations with Pearson's coefficient, and categorical data with chi-square tests. A binary logistic regression evaluated the effects of parenting style and feeding attitudes on breastfeeding beyond 2 years.

**RESULTS:**

While 98.7% of participants breastfed at least once, only 38.8% exclusively breastfed for the first 6 months, and 40.6% continued breastfeeding for 24 months or longer. It was found that a one-unit increase in the Iowa Infant Feeding Attitudes Scale score increased the probability of breastfeeding beyond 24 months by 5.4%, while each unit increase in the authoritarian attitude decreased it by 9.7%. Formula feeding duration was also positively correlated with authoritarian and overprotective parenting styles.

**CONCLUSION:**

Parenting styles and mothers’ infant feeding attitudes are two important factors affecting the rates of exclusive breastfeeding for the first 6 months and continuing breastfeeding beyond 24 months.

## INTRODUCTION

In 2012, the World Health Organization (WHO) set a goal for 50% of infants under 6 months to be exclusively breastfed by 2025^1^. Yet, most countries remain below these benchmarks. Marked disparities exist across high-income nations: exclusive breastfeeding rates at 6 months are ~1% in the United Kingdom, 13% in Denmark, 39% in the Netherlands, and 41% in Turkey^2^.

Multiple factors hinder the achievement of breastfeeding goals. Sociodemographic variables, such as maternal age, education, and socioeconomic status, strongly influence infant feeding, yet these factors are often difficult to alter^3,4^. Therefore, interventions should emphasize modifiable determinants. Targeted interventions and support services can substantially promote success, but first require identifying women who may choose not to breastfeed.

Maternal feeding attitudes have been extensively examined, with the Iowa Infant Feeding Attitudes Scale (IIFAS) validated across diverse contexts. However, prior studies primarily address feeding within the first 6–12 months^4–6^. Despite WHO recommendations to continue breastfeeding for 2 years or longer, to our knowledge, IIFAS has not yet been applied to extended breastfeeding durations.

Previous research has demonstrated an association between parenting styles and breastfeeding behaviors^7–9^. These parenting styles are categorized as democratic, authoritarian, permissive, and overprotective. It is essential to find answers to questions such as how parenting styles, especially aspects related to control and care, may affect or guide the decision to breastfeed or formula feed^8,10–12^. Studies in this area are few and typically cover the first 6 months or 1 year^8,9^. Despite WHO recommendations to continue breastfeeding for two years or longer, to our knowledge, we did not find any studies covering this period. Therefore, there is a need for research examining which parenting styles affect mothers’ feeding attitudes and behaviors, accounting for breastfeeding durations of 2 years or longer.

Despite WHO recommendations, breastfeeding rates for children 2 years of age and beyond remain very low^2^. Therefore, it is a priority for researchers and policymakers to identify the risk factors by determining the reasons for cessation or non-initiation of breastfeeding and to provide support to women who want to breastfeed their children. We wanted to investigate the question: Do parenting styles and infant feeding attitudes affect breastfeeding duration at 2 years of age and beyond?

### Purpose

In this study, we aimed to examine the relationships among breastfeeding duration through 2 years of age and beyond, and infant feeding attitudes and parenting styles, which are more flexible, "modifiable" factors for interventions.

## METHODS

This cross-sectional, correlational study was conducted at the Pediatrics and Social Pediatrics Outpatient Clinics of Akdeniz University Hospital between April 1, 2023, and April 1, 2024. Participants were mothers of healthy children aged 2–3 years attending routine follow-up. Eligibility required literacy in Turkish and effective communication, while exclusions applied to mothers or infants with breastfeeding-related medical conditions, psychiatric diagnoses, or prematurity.

### Calculating the sample size

The G*Power 3.1.9.4 program was used to calculate the sample size. In the sample article, the mean Parenting Styles Scale (PSS) scores for the two groups were 37.48±5.45 and 34.10±5.55, respectively, and the effect size was 0.61^13^. Using the data from this study, with an α error value of 0.05, a power (1-β error value) of 0.95, an effect size of 0.61, and an N1/2 value of 1, a total of 118 cases were needed for children aged two and over, with at least 59 individuals in the breastfed and non-breastfed groups. A total of 224 individuals were reached in the study: 91 breastfed children aged two and over, and 113 non-breastfed children.

### Measurement

Data were collected using four instruments: the Introductory Information Form, the Child Nutrition Questionnaire, the IIFAS, and the PSS.

The Introductory Information Form (18 items) assessed maternal sociodemographic and basic child characteristics. The Child Nutrition Questionnaire (nine items) evaluated children's nutritional status from birth.

IIFAS; the scale, developed by De La Mora and Russell and adapted to Turkish by Eksioğlu et al., measures women's attitudes toward breastfeeding and feeding method choice^5,14^. It consists of 17 items on a five-point Likert scale, with reverse scoring for formula-feeding items. Scores range from 17 to 85, with higher scores indicating a positive attitude toward breastfeeding. The Turkish version had a Cronbach Alpha of 0.71 in Eksioğlu et al.'s study^14^. In this study, Cronbach's α value was found to be 0.703.

PSS; the scale, developed in Turkish by Karabulut Demir and Şendil, assesses parenting attitudes of mothers and fathers with children aged 2–6. It consists of 46 items across four sub-dimensions, with scores ranging from 1 (never like this) to 5 (always like this)^10^. Higher scores indicate stronger adoption of the attitude in that dimension. Cronbach's alpha values in the study were 0.83 for the "democratic" dimension, 0.76 for "authoritarian," 0.75 for "overprotective," and 0.74 for "permissive." In this study, the following values were found to be 0.86, 0.82, 0.81, and 0.71, respectively. Mothers participating in the study were not categorized according to a single characteristic, but received one point for each characteristic identified by the PSS^10^.

### Data collection

Participants were informed about the study, and written consent was obtained prior to their participation. Data were collected face-to-face by the same physician in a private outpatient room to ensure comfort and confidentiality. Completion of the forms required approximately 15–20 min.

### Data analysis

Statistical analyses were conducted using Statistical Package for the Social Sciences 20.0. The Shapiro-Wilk test was employed to assess normality, and variables with skewness within ±1.5 were subjected to parametric tests. Group differences were evaluated using Independent Samples t-tests and analysis of variance, while correlations were determined through Pearson's correlation analysis. Categorical variables were analyzed using chi-square tests, with Pearson or Fisher's Exact tests applied as appropriate. Statistical significance was set at p<0.05. Binary logistic regression was utilized to examine the effects of parenting style and feeding attitudes on breastfeeding duration beyond 2 years.

## RESULTS

Of the 224 mothers who participated in the study, 70.5% were university graduates, and 41.5% were working. Among participants, 98.7% breastfed at least once. Of the children, 38.8% were exclusively breastfed for the first 6 months, and 23.7% were given water. After 6 months, 70.5% were introduced to complementary foods. Breastfeeding continued as follows: 83.5% between 6 and 12 months, 71% between 12 and 18 months, 54.9% between 18 and 24 months, and 40.6% for 24 months or more. Those exclusively breastfed for 6 months were 80% more likely to continue breastfeeding after 24 months (odds ratio: 1.80, 95%CI 1.313–2.466, p<0.0001).

The distribution of participants’ IIFAS and PSS mean scores according to breastfeeding characteristics is given in Table 1. The IIFAS mean score was higher in those breastfed for 6 months or more compared to those not breastfed (p<0.05). Additionally, those who received breast milk with supplementary food between 6 and 12 months had higher scores than those given formula (p<0.001). Those who exclusively breastfed for six months had lower authoritarian parenting scores (p=0.021). Those who gave water in the first 6 months had higher overprotective parenting scores (p=0.039). Mothers who breastfed for 24 months or more had lower authoritarian scores (p=0.011). Significant differences in overprotective parenting scores were found between feeding patterns between 6 and 12 months (p=0.033), with higher scores for those combining breast milk, formula, and complementary feeding. A significant difference in authoritarian scores was found after 24 months (p=0.013).

**Table 1:** Distribution of participants’ parenting style scale sub-dimension scores and Iowa Infant Feeding Attitudes Scale score averages by breastfeeding characteristics.

Categories		N/%	IIFAS Mean (SD)	p	Permissive Mean (SD)	p	Democratic Mean (SD)	p	Authoritarian Mean (SD)	p	Overprotective Mean (SD)	p
Exclusive breastfeeding for the first 6 months												
	Yes	87/38.8	67.51 (6.00)	**<0.001**	22.12 (4.60)	0.969	75.20 (6.47)	0.177	19.26 (4.67)	**0.021**	31.62 (6.18)	0.248
	No	137/61.2	64.47 (6.63)		22.14 (4.41)		73.85 (7.61)		20.93 (5.98)		32.61 (6.29)	
Provided water to the baby during the first 6 months												
	Yes	53/23.7	63.81 (6.49)	**0.019**	21.68 (4.50)	0.401	74.43 (8.26)	0.944	20.68 (5.78)	0.551	33.77 (6.89)	**0.039**
	No	171/76.3	66.22 (6.48)		22.27 (4.47)		74.35 (6.87)		20.15 (5.50)		31.74 (5.98)	
Breastfeeding status over 24 months												
	Yes	91/40.6	67.04 (5.69)	**0.006**	21.92 (4.37)	0.568	74.84 (7.38)	0.433	19.12 (4.86)	**0.011**	31.31 (6.56)	0.075
	No	133/59.4	64.70 (6.93)		22.27 (4.55)		74.06 (7.10)		21.06 (5.87)		32.84 (5.98)	
Feeding pattern between 6 and 12 months												
	B+CF (a)	152/67.9	66.86 (6.05)	**<0.001** difference (a–c)	21.93 (4.44)	0.478	74.48 (7.08)	0.767	19.84 (4.95)	0.256	31.70 (6.17)	**0.033** difference (a–b)
	B+F+CF (b)	35/15.6	64.51 (6.29)		22.29 (4.13)		75.06 (7.31)		21.79 (6.87)		34.56 (6.37)	
	F+CF (c)	32/14.3	61.91 (7.54)		23.13 (5.01)		73.41 (7.72)		20.91 (6.20)		27.80 (4.02)	
	CF (d)	5/2.2	61.00 (4.89)		20.60 (4.21)		72.80 (8.43)		19.20 (8.25)		32.23 (6.25)	
Feeding pattern over 24 months												
	B+CF (a)	90/40.4	67.12 (5.67)	**0.006** difference (a–c)	21.91 (4.40)	0.694	74.72 (7.35)	0.447	19.19 (4.84)	**0.013**	31.16 (6.44)	0.137
	F+CF (b)	22/9.8	62.64 (7.74)		22.82 (4.74)		72.55 (9.16)		22.82 (6.89)		33.23 (6.45)	
	CF (c)	111/49.8	65.11 (6.72)		22.16 (44.53)		74.36 (6.62)		20.71 (5.61)		32.76 (5.91)	

N: number of people; %: frequency; SD: standard deviation; p: significance level; IIFAS: Iowa Infant Feeding Attitude Scale; B: breastfeeding; F: formula; CF: complementary food. Statistically significant p-values are indicated in bold.

A low-level, significantly positive correlation was found between the participants’ IIFAS score and breastfeeding duration, as well as between the democratic parenting attitude score. Additionally, there was a low-level negative correlation between formula feeding duration and the overprotective parenting attitude score. A low-level positive relationship was also observed between formula feeding duration and both authoritarian and overprotective parenting attitude scores (Table 2).

**Table 2:** Correlation table some characteristics of the participants.

Variable		1	2	3	4	5	6	7	8
1. IIFAS									
	r (p-value)	1							
2. Breastfeeding duration/day*									
	r (p-value)	0.283 (<0.001)	1						
3. Feeding duration/day									
	r (p-value)	-0.233 (<0.001)	-0.607 (<0.01)	1					
4. Timing of complementary feeding**									
	r (p-value)	0.122 (0.068)	0.151 (0.024)	-0.081 (0.228)	1				
5. Permissive									
	r (p-value)	-0.068 (0.316)	-0.029 (0.664)	0.093 (0.169)	0.046 (0.497)	1			
6. Democratic									
	r (p-value)	0.221 (<0.001)	0.072 (0.291)	-0.035 (0.604)	-0.019 (0.784)	-0.077 (0.256)	1		
7. Authoritarian									
	r (p-value)	-0.023 (0.732)	-0.113 (0.094)	0.139 (0.039)	-0.045 (0.505)	0.191 (0.004)	-0.430 (<0.001)	1	
8. Overprotective									
	r (p-value)	-0.163 (0.015)	-0.087 (0.198)	0.150 (0.026)	-0.082 (0.227)	0.172 (0.010)	0.171 (0.010)	0.016 (0.817)	1

r: Pearson correlation coefficient; p: Level of significance; IIFAS: Iowa Infant Feeding Attitude Scale.

* Breastfeeding duration/day=Expresses the baby's breastfeeding duration in days (e.g., 90 days, 350 days, etc.).

** Timing of complementary feeding=It indicates the day on which complementary feeding was initiated (e.g., complementary feeding started on the 120th or 181st day of the baby).

Binary logistic regression analysis was conducted to assess the impact of parenting styles and infant feeding behaviors on breastfeeding status beyond 24 months. Maternal age, education level, family income, maternal employment status, and the presence of a sibling were included in the model and controlled for as potential confounding variables (Table 3). It was found that a one-unit increase in the IIFAS score increased the probability of breastfeeding beyond 24 months by 5.4%, while each unit increase in the authoritarian attitude decreased it by 9.7%.

**Table 3:** Evaluation of the effects of parenting styles and Iowa Infant Feeding Attitudes Scale score on breastfeeding rates in children aged 2 and over using binary logistic regression analysis.

Independent variables		N/%	Adjusted odds ratio (95%CI)	p
Mother's age			1.096 (1.02–1.17)	**0.008**
Mother's education level	High school or less	66/29.5	1*	0.893
	University	158/70.5	1.057 (0.47–2.37)	
Mother's working status	Not working	95/42.6	1*	
	Working	128/57.4	0.955 (0.48–1.89)	0.896
Perceived income level	Income is less than expenses	44/19.6	1*	0.669
	Balanced income and expenses	139/62.1	0.825 (0.37–1.79)	
	Income is more than expenses	41/18.3	1.160 (0.41–3.23)	
Sibling presence	No	106/47.3	1*	0.622
	Yes	118/52.7	1.174 (0.62–2.22)	
IIFAS score			1.054 (1.00–1.10)	**0.031**
Permissive score			1.021 (0.95–1.09)	0.570
Democratic score			0.982 (0.93–1.02)	0.443
Authoritarian score			0.903 (0.84–0.96)	**0.002**
Overprotective score			0.977 (0.92–1.03)	0.405

Nagelkerke R Square: 0.151.

* Reference group. p: level of significance; IIFAS: Iowa Infant Feeding Attitude Scale; CI: confidence interval. Statistically significant p-values are indicated in bold.

## DISCUSSION

Breast milk is the optimal infant nutrition, and its promotion remains a global public health priority^1^. This study, conducted in a baby-friendly hospital, found that while 98.7% of mothers initiated breastfeeding, continuation declined to 83.5% at 6 months, 71% at 12 months, and 40% at 24 months. Exclusive breastfeeding for 6 months reached 38.8%, exceeding rates in some European countries yet remaining below WHO targets^1,2^. These figures should also be interpreted cautiously, as the sample included only healthy mothers and infants.

Consistent with prior research, maternal attitudes were strongly associated with breastfeeding during the first 6 months^3,6^. Beyond this period, we found that mothers who breastfed for 6–24 months or longer had significantly higher IIFAS scores than mothers who did not breastfeed at all. We also showed that each unit increase in IIFAS value increased the probability of breastfeeding at age 2 and beyond by 5.4%. In our study, higher IIFAS scores were associated with longer breastfeeding and reduced formula use. These results align with prior evidence linking positive maternal attitudes to breastfeeding initiation and duration, while also contributing novel data on practices extending past 2 years^6,15^.

Parenting styles have also been associated with breastfeeding attitudes^8,9,16^. In their study involving infant-mother pairs in the first year of life, Brown and Arnott showed that a high level of parent-led routine reduced the likelihood of a mother initiating and continuing breastfeeding^8^. In the study conducted by Davis et al., it was shown that higher scores in the "permissive" and "authoritative/competent" dimensions were significantly associated with more positive attitudes toward breastfeeding, and higher scores in the "uninvolved" and "authoritarian" dimensions were associated with less positive attitudes^9^.

This study extends the literature from 1 to 2 years to examine the impact of parenting styles on breastfeeding duration beyond 2 years. We found that each unit increase in authoritarian parenting style decreased the likelihood of breastfeeding beyond 2 years by 9.7%, while each unit increase in IIFAS score increased it by 5.4%. There was also a positive association between breastfeeding duration and IIFAS scores and a democratic parenting style, and a negative association with an overprotective parenting style.

In thıs study, mothers who did not exclusively breastfeed their babies for the first 6 months had higher authoritarian parenting style scores than mothers who did. We found that mothers who gave their babies water and formula in addition to breastmilk had higher overprotective parenting style scores. The relationship between parenting styles and breastfeeding attitudes found in this study is consistent with studies in this field^8,9,16^.

It is not easy to establish a direct relationship between parenting styles and breastfeeding and breastfeeding duration. Breastfeeding for a long time may affect the bond between mother and baby and may change the parenting style, and it has been noted that mothers with a certain parenting style may show variability in breastfeeding and breastfeeding duration^8,11,12,16^.

Our study results are consistent with the literature showing that modifiable factors such as mothers’ infant feeding attitudes and parenting styles are associated with breastfeeding duration and contribute to the knowledge in this field by providing data for 6 months later^3,6,8,9^.

These findings may guide health professionals in prenatal and postnatal care by linking parenting styles with breastfeeding attitudes, enabling early identification of mothers requiring additional counseling. Recognizing mothers with low infant feeding attitude scores and authoritarian or overprotective parenting styles allows health providers, such as midwives, nurses, and doctors, to offer personalized guidance and solutions to meet each mother's specific needs.

### Strengths and weaknesses of the study

This study has several limitations. First, it included only mothers of children aged 2–3 years, and retrospective questions about birth and infant feeding may have introduced recall and social desirability biases. However, there is evidence that maternal recall of breastfeeding practice up to 3 years is valid and reliable^17^. Second, the cross-sectional design limits causal inference and does not capture long-term changes in parenting attitudes. Despite these limitations, the study provides valuable insight into the relationship between parenting attitudes and breastfeeding through 2 years, offering a basis for future longitudinal research.

In future studies, health practitioners can design interventions for mothers who may feel negatively about breastfeeding by understanding the determinants of positive attitudes toward breastfeeding. A practical approach is to clarify mothers’ questions about breastfeeding by assessing infant feeding attitudes, parenting style, and expected breastfeeding duration. These interventions may facilitate breastfeeding initiation and increase breastfeeding rates through age 2, thereby improving overall maternal and infant health.

## CONCLUSION

Parenting styles and infant feeding attitudes are two important factors affecting exclusive breastfeeding rates for the first 6 months and continuing breastfeeding beyond 24 months, which are the WHO targets. Identifying mothers with low infant feeding attitude scale scores and authoritarian or overprotective parenting styles can contribute to the individualization of information provided by health professionals, such as midwives, nurses, and doctors, who offer counseling and the ability to provide solutions to the mother's problems.

## Data Availability

The datasets generated and/or analyzed during the current study are available from the corresponding author upon reasonable request.
